# Relationship between Kt/V urea-based dialysis adequacy and nutritional status and their effect on the components of the quality of life in incident peritoneal dialysis patients

**DOI:** 10.1186/1471-2369-13-39

**Published:** 2012-06-14

**Authors:** Jin-Bor Chen, King-Kwan Lam, Yu-Jen Su, Wen-Chin Lee, Ben-Chung Cheng, Chien-Chun Kuo, Chien-Hsing Wu, Eton Lin, Yi-Chun Wang, Te-Chuan Chen, Shang-Chih Liao

**Affiliations:** 1 Division of Nephrology, Kaohsiung Chang Gung Memorial Hospital and Chang Gung University College of Medicine, Kaohsiung, Taiwan; 2 Division of Nephrology, Chang Gung Memorial Hospital, Kaohsiung, No. 123, Ta Pei Road, Niao Sung District, Kaohsiung City, Taiwan

**Keywords:** Peritoneal dialysis, Quality of life, SF-36

## Abstract

**Background:**

It is well known that the quality of life of patients with chronic kidney disease can be improved by dialysis. While previous studies have used retrospective designs and adhered to a standard target prescribed by clinical guidelines, our study prospectively investigates the association between the adequacy of peritoneal dialysis (PD) and measures of nutritional status on quality-of-life domains in a cohort of incident PD patients.

**Methods:**

It was a prospective 6-month observational study. Eighty incident PD participants who were treated in a hospital-based PD center were enrolled. The period of enrollment was January 2009–June 2010; follow-up continued until December 2010. PD adequacy indices, including Kt/V urea, weekly Ccr (WCcr), measures of nutritional status (albumin, BMI), and nPCR were measured at 1 month and 6 months after PD initiation. SF-36 health survey questionnaires were used to measure the quality of life. The outcomes were used to measure the changes in the domains of the SF-36 after 6 months of PD therapy.

**Results:**

Seventy-seven incident patients who underwent PD for 6 months were included in the study. The mean age was 47.3 years, and the male-to-female ratio was 38:39. A peritoneal Kt/V urea value of 1.2, which was also the baseline cutoff value, was found to have the highest influence on SF-36 domains. Patients with baseline peritoneal Kt/V urea value of <1.2 showed improvement in the physical functioning and role limitation of physical functioning components after 6 months of PD. In contrast, patients with baseline peritoneal Kt/V urea values of ≥1.2 showed remarkable improvement in the general health, physical functioning, role limitation caused by physical problems, and bodily pain components. However, the trend of improvement decreased in patients with baseline nPCR of <1.2. Baseline renal WCcr did not influence the improvement in the SF-36 domains.

**Limitations:**

A small cohort and a short observation period.

**Conclusions:**

The baseline level of peritoneal Kt/V urea affected the components of the quality of life after PD initiation. In contrast, a lower baseline nPCR level was associated with deterioration in the quality of life after PD therapy.

## Background

The concept of health-related quality of life (HRQoL) in patients with chronic kidney disease (CKD), including end-stage renal disease (ESRD), has evolved since the inception of renal replacement therapy, evolving from ensuring easy survival to achieving a sense of well-being [1]. Patients with CKD tend to show a reduction in their quality of life (QoL) because of the restrictions resulting from CKD treatment. Multiple factors, such as the presence of co-morbidities, can further reduce the QoL. Interventions aiming to improve the clinical condition and the QoL of these patients are of paramount importance, since the latter is directly associated with mortality. The association between the reduction of QoL and preventable and controllable factors including diabetes [2], old age [3], inadequacy of dialysis, inflammation and poor nutrition, is still unclear. In addition, studies have shown that dialysis initiation improves the QoL in ESRD patients [4-8]. However, the association between dialysis adequacy indices or nutritional parameters and QoL is inconsistent. These results may be inconsistent owing to the small sample of patients in those studies, non-ESRD-specific assessments, inadequate observation periods, or other factors.

Several validated, disease-specific HRQoL questionnaires can be used for the dialysis cohort, such as the World Health Organization Quality of Life Survey (WHOQOL), Short Form (SF)-36 health survey, the Kidney Disease Quality of Life (kDQOL), and the Choices Health Experiences Questionnaire (CHEQ). Patients with Kt/V urea values above 2.0 undergoing peritoneal dialysis (PD) were found to have higher total SF-36 scores than patients with Kt/V urea values below 2.0 [6], although some researchers argue that the correlation between Kt/V urea values and SF-36 scores is not significant [8]. There are only 2 validated native-language editions of questionnaires in Taiwan: the WHOQOL and the SF-36. In Taiwan, the SF-36 questionnaires have been used as a validation tool in a multi-center study comparing the QoL between PD and hemodialysis patients [9].

In this study, we used the SF-36 to assess the QoL in an incident cohort of PD patients. The purpose of the study was to investigate which components of the SF-36 could be improved after PD initiation. In addition, the influences of PD adequacy indices and nutritional status measures on the components of the SF-36 were explored.

## Methods

Incident ESRD patients who consulted a PD institution at a medical center in southern Taiwan between January 2009 and June 2010 were enrolled in this prospective study. The following were the inclusion criteria: (1) new PD patients; (2) aged above 17 years; (3) able to express themselves; (4) no history of psychiatric disease; and (5) clinically stable with no evidence of chronic or acute infections, inflammatory disorders, malignancy, or anti-inflammatory drug use 3 months prior to enrollment. The exclusion criteria were as follows: (1) aged below 17 years; (2) unable to receive PD therapy for three months after PD initiation; (3) unable to complete the questionnaires by themselves; (4) major clinical events requiring hospital admission; and (5) discontinuation of PD owing to kidney transplantation, technique failure, death, transfer to hemodialysis, or loss to follow-up. As per the study protocol requirement, all patients had completed at least 6 months of consecutive PD therapy, and 77 of 80 clinically stable patients (38 men and 39 women) were finally eligible. The mean age was 47.3 years. All the patients were dialyzed using commercially available dialysate (pH 5.2; Dianeal PD solution; Baxter, Singapore) containing 40 mmol/L lactate. Forty-five patients received continuous ambulatory PD (CAPD) therapy, with 4 exchanges every day. Thirty-two patients received automated PD (APD) therapy.

The QoL was measured using the Chinese version of the Short Form (SF)-36 health survey questionnaire (Taiwan Standard Version 1.0)—a generic self-report QoL instrument—comprising 36 items, which are assigned to the following 8 domains: general health, physical functioning, role limitation due to physical problems, bodily pain, mental health, social functioning, role limitation due to emotional problems, and vitality. The first 4 domains constitute the physical component scale, and the next 4 constitute the mental component scale. Higher scores indicate better QoL.

All the patients were asked to complete the SF-36 questionnaire prior to PD therapy as well as 6 months after PD initiation. A standard peritoneal equilibrium test (PET) was performed in the first month and then after 6 months after PD initiation. The clinical characteristics of all patients, including demographic and biochemical data, PD adequacy indices of renal and peritoneal Kt/V urea and creatinine clearance (Ccr), and nutritional indices (serum albumin, normalized protein catabolic rate [nPCR]) were collected as baseline information at the beginning of the study. These data were collected again at 6 months for statistical analysis.

The protocol for the study was approved by the Committee on Human Research at Kaohsiung Chang Gung Memorial Hospital (CMRPG880091) and conducted in accordance with the Declaration of Helsinki. All participants signed an informed consent form before taking part in the study.

### Statistical analysis

Using a general linear model, we determined an applicable Kt/V value to determine the effect of Kt/V on the QoL (Table 1). On the basis of the Kt/V value selected by us, the following patient characteristics were recorded, grouped, and compared using the Mann–Whitney test, Chi-square test, or likelihood ratio test: age; gender; clinical data including values for peritoneal urea Kt/V, residual renal urea Kt/V, total Kt/V, weekly peritoneal Ccr, weekly residual renal Ccr, total weekly Ccr, high-sensitivity C-reactive protein (hs-CRP), albumin, GPT, hemoglobin (Hb), and nPCR; and the 8 multi-item domains of the SF-36. Thus, age, peritoneal Kt/V, residual renal WCcr, and nPCR were regarded as independent variables for exploring the effect of PD therapy on SF-36 scores. Statistical analyses were performed using the IBM SPSS Statistics 19 (SPSS Inc. Chicago, IL) program.

**Table 1:** The influence of stratified peritoneal Kt/V urea levels on SF-36 domains

**SF-36 Domains**	**Physical functioning**		**Role limitation - physical**		**Bodily pain**		**General health**		**Vitality**		**Social functioning**		**Role limitation - emotional**		**Mental health**	
	***β***	***P*-value**	***β***	***P*-value**	***β***	***P*-value**	***β***	***P*-value**	***β***	***P*-value**	***β***	***P*-value**	***β***	***P*-value**	***β***	***P*-value**
Peritoneal Kt/V urea																
<1.2	18.4	0.074	13.59	0.224	21.4	0.078	31.75	0.039	4.51	0.407	-3.65	0.814	3.51	0.754	4.97	0.440
1.2≦Kt/V<1.3	27.62	0.015	27.27	0.027	5.46	0.676	37.49	0.025	8.10	0.174	6.47	0.702	14.95	0.224	11.18	0.113
1.3≦Kt/V<1.4	24.92	0.016	27.39	0.016	22.7	0.061	37.47	0.015	5.94	0.274	7.84	0.612	17.80	0.115	7.64	0.235
1.4≦Kt/V<1.5	20.71	0.047	24.73	0.030	25.04	0.042	27.91	0.070	9.94	0.073	11.77	0.452	10.81	0.341	10.64	0.104
1.5≦Kt/V<1.6	4.98	0.678	14.64	0.266	25.51	0.075	12.28	0.491	-1.53	0.811	-14.57	0.426	0.70	0.957	4.14	0.585
≧1.6	20.91	0.028	18.96	0.067	19.83	0.076	43.74	0.002	10.59	0.037	6.66	0.641	12.33	0.235	7.55	0.204

Statistical method: general linear model.

## Results

### Association between the baseline peritoneal kt/V urea values and the scores of the SF-36 components

More components of the SF-36 were influenced by peritoneal Kt/V values when the cutoff peritoneal Kt/V value was set at 1.2. General health, physical functioning, role limitation due to physical problems, and bodily pain scores had significantly improved when the peritoneal Kt/V value was above 1.2. In contrast, the scores for only the general health component improved when the peritoneal Kt/V value was below 1.2 (Table 1).

### Stratification of the patients on the basis of peritoneal kt/V urea and nPCR values

The patients were stratified into 2 groups on the basis of baseline Kt/V urea values above or below 1.2. The peritoneal Kt/V urea, total Kt/V urea, and weekly peritoneal Ccr values (Table 2) were significantly different between both groups. When the patients were stratified according to an nPCR value above or below 1.2, those with nPCR values of ≥1.2 had higher total Kt/V urea and WCcr values than those with nPCR values of <1.2 (Table 3). The baseline scores for the SF-36 components did not differ between the groups stratified on the basis of cutoff values of 1.2 of Kt/V urea or nPCR values (Tables 2 and 3)..

**Table 2:** Baseline patient characteristics and SF-36 domains stratified by peritoneal Kt/V urea levels

	**Peritoneal Kt/V urea**		
	**<1.2 (n = 11)**	**≧1.2 (n = 66)**	
	**Median (Range)**	**Median (Range)**	***P***
Age (years)	54.3 (19.9 - 68.1)	48.8 (17.6 - 80.1)	1
Gender, Male (n)	5	33	0.780*
DM, n (%)	2 (18.2)	20 (30.3)	0.392^a^
BMI	20.1(14.4 – 28.2)	20.5(16.4 – 35.2)	0.760
PD modality, CAPD/APD (n)	8/3	34/32	0.247*
Hb (g/dL)	8.9 (3 - 13)	8.9 (6 - 12)	0.708
Albumin (g/dL)	3.6 (2.3 - 4.2)	3.6 (2.4 - 4.9)	0.86
GPT (U/L)	12 (4 - 41)	15 (3 - 458)	0.676
Cr (mg/dL)	12.4 (5.1 - 21.7)	12 (3.9 - 20.3)	1
hs-CRP (mg/L)	3.79 (0.2 - 40.1)	4.8 (0.2 - 199)	0.067
Peritoneal Kt/V urea	1.01 (0.7 - 1.2)	1.44 (1.2 - 2.3)	<0.001
Residual renal Kt/V urea	0.79 (0.2 - 4.1)	0.54 (0 - 2.1)	0.111
Total Kt/V urea	1.78 (1.1 - 4.8)	2.11 (1.2 - 3.3)	0.003
Peritoneal WCcr L/week/1.73m^2^	31.5 (10.9 - 40.2)	37.1 (20.5 - 55.2)	<0.001
Residual renal WCcr L/week/1.73m^2^	38.6 (8.8 - 262.4)	29.8 (0 - 204.8)	0.103
Total WCcr L/week/1.73m^2^	68.2 (44.9 - 348.4)	72.7 (31.4 - 226.7)	0.58
nPCR	0.96 (0.5 - 1.5)	1.07 (0.5 - 1.6)	0.067
Physical functioning	76.7 (43.3 - 100)	76.7 (33.3 - 100)	0.727
Role limitation - physical	65.4 (46.2 - 100)	61.5 (38.5 - 100)	0.931
Bodily pain	63.6 (27.3 - 100)	72.7 (27.3 - 100)	0.172
General health	35 (30 - 90)	40 (20 - 100)	0.148
Vitality	31.5 (11.1 - 46.3)	35.2 (7.4 - 53.7)	0.676
Social functioning	50 (20 - 100)	60 (20 - 100)	0.537
Role limitation - emotional	63.6 (54.5 - 100)	72.7 (36.4 - 100)	0.382
Mental health	27.5 (20 - 45)	30 (5 - 50)	0.811

* chi-square test, ^a^ likelihood ratio test.

**Table 3:** Baseline patient characteristics and SF-36 domains stratified by nPCR levels

	**nPCR**		
	**<1.2 (n = 55)**	**≧1.2 (n = 22)**	
	**Median (Range)**	**Median (Range)**	***P***
Age (years)	50.7 (17.6 - 80.1)	47.5 (19.9 - 68.1)	0.665
Gender, Male (n)	5	33	0.780*
DM, n (%)	2 (18.2)	20 (30.3)	0.392^a^
BMI	24.4 (16.4 – 35.2)	20.6 (14.4 – 25.8)	<0.001
PD modality, CAPD/APD (n)	26/29	16/6	<0.01 *
Hb (g/dL)	9 (3 - 13)	8.8 (6 - 11)	0.388
Albumin (g/dL)	3.6 (2.4 - 69)	3.5 (2.3 - 4.7)	0.663
GPT (U/L)	15 (3 - 458)	13.5 (7 - 39)	0.885
Cr (mg/dL)	11.5 (5.4 - 21.7)	12.6 (3.9 - 20.1)	0.564
hs-CRP (mg/L)	3.33 (0.2 - 199)	7.86 (0.5 - 65.2)	0.299
Peritoneal Kt/V urea	1.36 (0.7 - 2.3)	1.43 (0.7 - 2.2)	0.051
Residual renal Kt/V urea	0.54 (0 - 1.44)	0.91 (0 - 4.1)	0.009
Total Kt/V urea	1.92 (1.1 - 3.0)	2.34 (1.8 - 4.8)	<0.001
Peritoneal WCcr L/week/1.73m^2^	35.3 (20.9 - 55.2)	36 (10.9 - 52.1)	0.973
Residual renal WCcr L/week/1.73m^2^	29 (0 - 204.8)	39.2 (0 - 262.4)	0.167
Total WCcr L/week/1.73m^2^	68.8 (31.4 - 226.7)	84.4 (45.6 - 348.4)	0.007
nPCR	0.96 (0.5 - 1.2)	1.35 (1.2 - 1.6)	<0.001
Physical functioning	76.7 (33.3 - 100)	78.3 (36.7 - 100)	0.612
Role limitation - physical	61.5 (38.5 - 100)	65.4 (46.2 - 100)	0.769
Bodily pain	72.7 (27.3 - 100)	72.7 (27.3 - 100)	0.659
General health	40 (20 - 100)	35 (20 - 90)	0.416
Vitality	35.2 (7.4 - 51.9)	34.3 (11.1 - 44.4)	1
Social functioning	60 (20 - 100)	60 (20 - 80)	0.257
Role limitation - emotional	68.2 (36.4 - 100)	68.2 (45.5 - 100)	1
Mental health	30 (10 - 50)	30 (5 - 45)	0.938

* chi-square test, ^a^ likelihood ratio test

### The effects of baseline peritoneal kt/V urea, renal WCcr, and nPCR values on the scores of the SF-36 components after 6 months of PD

The patients who had baseline peritoneal Kt/V urea values of <1.2 showed an improvement in physical functioning and role limitations due to physical problems after 6 months of PD therapy. Patients who had baseline peritoneal Kt/V urea of ≥1.2 showed improvement in general health, physical functioning, role limitation due to physical problems, and bodily pain (Table 4). Residual renal function of either <40 L·week^-1^·1.73 m^-2^ or ≥40 L·week^-1^·1.73 m^-2^ did not have an impact on SF-36 scores after 6 months of PD therapy. Deterioration was observed in all the components of the SF-36 after 6 months of PD therapy in patients with baseline nPCR values of <1.2 (Table 4).

**Table 4:** Multivariate analysis using the general linear model to assess the difference of influence on the SF-36 between one month and six months following peritoneal dialysis therapy

	**Physical functioning**			**Role limitation –physical**			**Bodily pain**			**General health**		
	**β**	**(CI)**	***P***	**β**	**(CI)**	***P***	**β**	**(CI)**	***p***	***β***	**(CI)**	***p***
Age	-0.309	(-0.67-0.05)	0.09	-0.357	(-0.75-0.03)	0.07	-0.24	(-0.66-0.18)	0.262	-0.266	(-0.79-0.26)	0.32
Peritoneal Kt/V												
<1.2	26.971	(3.81-50.14)	0.023	34.326	(9.21-59.44)	0.008	25.518	(-1.88-52.91)	0.067	30.703	(-3.53-64.94)	0.078
≧1.2	21.166	(1.64-40.70)	0.034	26.026	(4.85-47.20)	0.017	23.245	(-0.15-46.34)	0.049	33.373	(4.51-62.24)	0.024
Residual renal WCcr												
<40	2.824	(-7.24-12.89)	0.578	-4.666	(-15.58-6.25)	0.397	-3.383	(-15.29-8.52)	0.573	10.481	(-4.40-25.36)	0.164
≧40	0	--		0	--		0	--		0	--	
nPCR												
<1.2	-5.645	(-16.25-4.96)	0.292	-3.325	(-14.82-8.17)	0.566	-0.083	(-12.62-12.46)	0.99	-9.164	(-24.83-6.51)	0.247
≧1.2	0	--		0	--		0	--		0	--	
	**Vitality**			**Social functioning**			**Role limitation- emotional**			**Mental health**		
	***β***	**(CI)**	***p***	***β***	**(CI)**	***P***	***β***	**(CI)**	***P***	***β***	**(CI)**	***p***
Age	-0.089	(-0.28-0.10)	0.346	0.107	(-0.42-0.63)	0.687	-0.154	(-0.55-0.24)	0.437	-0.142	(-0.37-0.08)	0.206
Peritoneal Kt/V												
<1.2	11.974	(-0.21-24.16)	0.054	6.724	(-27.39-40.83)	0.695	19.574	(-5.92-45.07)	0.13	11.234	(-3.18-25.65)	0.125
≧1.2	9.276	(-1.0-19.55)	0.076	2.663	(-26.09-31.42)	0.854	15.297	(-6.20-36.79)	0.16	8.402	(-3.75-20.55)	0.172
Residual renal WCcr												
<40	2.936	(-2.36-8.23)	0.273	13.906	(-0.92-28.73)	0.066	-0.775	(-11.86-10.31)	0.889	0.949	(-5.32-7.21)	0.763
≧40	0	--		0	--		0	--		0	--	
nPCR												
<1.2	-6.484	(-12.06- -0.91)	0.023	-13.46	(-29.07-2.15)	0.09	-6.162	(-17.83-5.51)	0.296	-2.398	(-9.0-4.20)	0.471
≧1.2	0	--		0	--		0	--		0	--	

Multivariate analysis:

## Discussion

Small solute clearance measured by Kt/V urea is known to be one of the major determinants of dialysis adequacy. A growing body of evidence has suggested that a strong link exists between Kt/V urea values and mortality rates in dialysis populations [10]. Therefore, to reduce the risk of mortality and improve dialysis adequacy, the total (renal + peritoneal) Kt/V urea value should not be less than 1.7 for small solute removal, according to the guidelines of the Kidney Disease Outcomes Quality Initiative (KDOQI) and the International Society for Peritoneal Dialysis (ISPD) [11,12]. To reach this target, however, patients might experience adverse effects, including hernias (from the increased intra-abdominal pressure due to large volumes of dialysis solution), weight gain, and other metabolic consequences due to increased exposure to glucose. Furthermore, the increased amount of time needed to perform the exchanges is less acceptable to patients. These factors might not only adversely affect the willingness of the patients to continue PD therapy but also affect the QoL in incident PD patients. However, one study reported that a lower Kt/V urea target was associated with a similar survival rate [13], leading us to consider what the impact of a lower Kt/V urea target would be on QoL. In our study, we carefully stratified our patients on the basis of peritoneal Kt/V urea values into 2 groups. Peritoneal Kt/V urea is the major component of total weekly Kt/V urea and is the critical component targeted in dialysis regimens. We found that patients with a peritoneal Kt/V urea value of >1.2 experienced significant improvement in the QoL, especially in the domains of general health, physical function, and role limitation due to physical problems, at 6 months after the initiation of PD. Our results suggest that PD on its own is highly beneficial in improving the QoL in patients whose peritoneal Kt/V urea value is above 1.2. This result implies that some domains of the QoL could be improved after 6 months of PD therapy with a lower peritoneal Kt/V urea target. However, the influence of a lower peritoneal Kt/V urea value on long-term QoL and patient survival needs to be further investigated. Our study also suggests that it might not be necessary to pursue a higher adequacy target at the expense of QoL in the early phase of PD initiation. By not pursuing a higher adequacy target, the early discontinuation of incident PD patients observed occasionally can be avoided.

The nPCR value, also called protein equivalent of nitrogen appearance (PNA), can be used to assess dietary protein intake in patients who are in a steady state. It has also been adopted as a reference value to adjust dialysis prescription. When poor nutrition (e.g., nPCR < 0.8 g·kg^-1^·day^-1^) or inadequate dialysis (e.g., Kt/V urea < 1.2) is apparent, the dialysis prescription has to be adjusted to meet the clinical situation. Moreover, to ascertain whether a significant change has occurred may require several months of nPCR and Kt/V urea level monitoring. A previous study has demonstrated that mortality rates increased in dialysis patients with nPCR values of less than 0.8 g·kg^-1^·day^-1^ or greater than 1.4 g·kg^-1^·day^-1^[14]. Among patients with nPCR levels between 0.8 g·kg^-1^·day^-1^ and 1.2 g·kg^-1^·day^-1^, an increase or decrease in protein intake during the first 6 months was associated with an increase or decrease in the survival rate over the subsequent 18 months, respectively. Thus, reduced survival is associated with an initially low nPCR and decreased protein intake over time. In the present study, patients with baseline nPCR values of >1.2 g·kg^-1^·day^-1^ had higher renal Kt/V urea values and higher weekly Ccr values than those with nPCR values of <1.2 g·kg^-1^·day^-1^. Patients with nPCR values greater than 1.2 g·kg^-1^·day^-1^ showed improvement in SF-36 domain scores after 6 months of PD therapy. In contrast, patients with nPCR values of <1.2 g·kg^-1^·day^-1^ showed a decrease in SF-36 domain scores after 6 months of PD therapy. The exact mechanism by which residual renal function improves the QoL in subjects with nPCR values of >1.2 g·kg^-1^·day^-1^ needs to be further investigated. However, this finding implicates nutritional status as key factor in QoL improvement with PD initiation. Therefore, it is essential to devise a strategy to increase the nutritional status primarily in the early phase of PD commencement.

In the past few years, some investigators have focused on the reasons for the early discontinuation PD therapy by patients. The NECOSAD study demonstrated that the psychosocial effect of PD therapy was a one of the major reasons why patients discontinued therapy in the first 3 months [15]. Another study from a single PD center also showed that psychosocial effects were a major cause of early discontinuation of therapy in the first 6 months of PD therapy [16]. However, one study has shown that the measures of social relationships and physical health of PD patients did not significantly change during the early and late phases of treatment [17]. Furthermore, it has been found in one study that emotional defensiveness affects the physical and mental components of HRQoL in patients undergoing dialysis [18]. In that study, the incident PD patients needed an adjustment period to adapt to their uncertain psychological status; the complex, time-consuming PD exchange procedure affected their response to PD therapy. This finding suggests that psychological effects must not be ignored in the management of patients undergoing dialysis. Our study clearly showed that PD could improve the QoL, as measured by the SF-36 instrument, after 6 months of therapy. However, we found that a lower peritoneal Kt/V urea value further contributed to a remarkable improvement in the QoL. We assume that a more satisfactory PD prescription might reduce the psychosocial burden on incident PD patients, even though it would attain a lower adequacy index. This approach did not sacrifice the QoL in incident PD patients, as per our results.

## Conclusion

In summary, the QoL in incident PD patients can be improved with a low of Kt/V urea value in the PD prescription. Moreover, lower nPCR levels will offset the benefits of PD initiation on the QoL improvement. Additionally, a few aspects of the QoL domains in the SF-36 could be further improved in 6-month PD therapy in the cohort with low Kt/V urea levels. The results could guide medical staff in individualizing the medical plan for incident PD patients. We conclude that a stepwise PD program is essential for incident PD patients. This gradual approach to PD prescription need not be at the expense of QoL in incident PD patients.

## Abbreviations

APD, Automated Peritoneal Dialysis; CAPD, Continuous Ambulatory Peritoneal Dialysis; Ccr, Creatinine Clearance; CHEQ, Choices Health Experiences Questionnaire; CI, Confidence Interval; CKD, Chronic Kidney Disease; ESRD, End-stage Renal Disease; hs-CRP, High-sensitivity C-reactive Protein; kDQOL, Kidney Disease Quality of Life; nPCR, Normalized Protein Catabolic Rate; PD, Peritoneal Dialysis; PET, Peritoneal Equilibrium Test; PNA, Protein Equivalent of Nitrogen Appearance; QoL, Quality of Life; SF-36, Short Form Health Survey-36; WHOQOL, World Health Organization Quality of Life Survey; WCcr, Weekly Creatinine Clearance.

## Competing interests

The authors declare that they have no competing interests.

## Authors’ contributions

JBC and KKL collected and interpreted the data, critically reviewed the manuscript, and drafted the manuscript after incorporating some revisions. YJS conceived the research question, interpreted the data, and critically reviewed the manuscript. WCL, BCC, CCK, and YCW participated in the acquisition and interpretation of data, revised the manuscript critically, and performed the statistical analyses. CHW and EL assisted with data collection, interpreted the data, and were involved in the drafting of the manuscript. TCC and SCL conceived, designed, and coordinated the study and commented on the final draft. All authors have read and approved the final manuscript.

## Author details

All authors worked at the Division of Nephrology, Kaohsiung Chang Gung Memorial Hospital and Chang Gung University College of Medicine, Kaohsiung, Taiwan.

## Pre-publication history

The pre-publication history for this paper can be accessed here:

http://www.biomedcentral.com/1471-2369/13/39/prepub
